# Effects of orange peel extract (*Citrus sinensis*) treatment on zebrafish oocytes (*Danio rerio*) exposed to heat stress

**DOI:** 10.14202/vetworld.2024.1821-1827

**Published:** 2024-08-20

**Authors:** Gretania Residiwati, Almira Ghina Shalawati, Muhamad Arfan Lesmana, Agri Kaltaria Anisa, Bonick Kartini Lonameo, Habib Syaiful Arif Tuska

**Affiliations:** 1 Laboratory of Embryology, Faculty of Veterinary Medicine, Universitas Brawijaya, Malang-Indonesia; 2 Laboratory of Veterinary Surgery and Radiology, Faculty of Veterinary Medicine, Universitas Brawijaya, Malang-Indonesia; 3 Laboratory of Veterinary Pharmacology, Faculty of Veterinary Medicine, Universitas Brawijaya, Malang-Indonesia; 4 Laboratory of Veterinary Reproduction, Faculty of Veterinary Medicine, Universitas Brawijaya, Malang-Indonesia

**Keywords:** heat stress, orange peel extract, Zebrafish oocytes

## Abstract

**Background and Aim:**

Heat stress (HS) can negatively impact oocytes by disrupting mitochondrial activity, increasing the production of reactive oxygen species, and decreasing antioxidant levels. This study investigated the impact of orange peel (OP) exposure on zebrafish oocytes (ZOs) diameter, survival rate, and germinal vesicle breakdown (GVBD) during HS.

**Material and Methods:**

We investigated the antioxidant effect of flavonoids (concentration = 328.58 ppm) derived from OP (*Citrus sinensis*) added to *in vitro maturation* (IVM) media of ZOs (*Danio rerio*) under non-heat stress (NHS) and HS conditions to mimic *in vivo* HS conditions due to the global warming phenomenon on females. ZO in stage 3 (n = 1080) was treated with 4 μL of OP extract (not treated/control) under HS: 32°C (Heat stress 32°C solution/Heat stress 32°C orange peel [HS32S/HS32O]) and 34°C (Heat stress 34°C solution/Heat stress 34°C orange peel [HS34S/HS34O]); and NHS: 28°C (Non-heat stress solution/Non-heat stress orange peel [NHSS/NHSO]), during maturation. After 24 h of maturation, we observed the oocyte diameter, survival rate, and GVBD rate. The data were analyzed with IBM Statistics 23 software using two-way analysis of variance and Kruskal–Wallis (p < 0.05).

**Results:**

The highest oocyte diameter data were in NHS treated with OP extract (NHSO) group (0.759 ± 0.01; mean ± standard error) compared with HS group using and without OP extract (HS32S [0.583 ± 0.02]; HS32O [0.689 ± 0.02]; HS34S [0.554 ± 0.02]; and HS34O [0.604 ± 0.02]). The survival rate of OP treated group, namely, NHSO (93% ± 3%), HS32O (85% ± 2%), and HS34O (80% ± 2%) was higher than that of the group without treatment (NHSS [83% ± 3%], HS32S [71% ± 6%], and HS34S [63% ± 3%]). ZO treated with OP extract (NHSO [93% ± 3%], HS32O [85% ± 2%], and HS34O [80% ± 2%]) showed a higher GVBD rate than the group without treatment (NHSS [83% ± 3%], HS32S [71% ± 6%], and HS34S [63% ± 3%]).

**Conclusion:**

It revealed that OP can enhance the oocyte diameter, survival rate, and GVBD rate of ZO under NHS and HS. Further investigation should be conducted to determine the effect of OP extract (*C. sinensis*) on *in vivo* conditions in females as an alternative treatment to face global warming.

## Introduction

Since the Earth gave a crucial sign with 2.45°F (1.36°C) warmer in 2023 than in the late 19^th^ century (1850–1990) [1], global warming has become one of today’s priority challenges. Global warming manifests in the earth’s ecosystem, which is unbalanced due to the process of increasing the average temperature of the earth’s atmosphere impacted by heat stress (HS), which affects the physiology, morphology, anatomy, and reproduction of humans and animals [2–4].

HS refers to the physiological consequences of body temperature elevation resulting from combined internal and external stimuli [5]. According to Residiwati *et al*. [6], HS affected the kinetic parameters, DNA, acrosome, plasma membrane, and mitochondrial activity of sperm. The impact of HS (40.5°C) on bovine oocytes and embryos affected various developmental parameters; however, lycopene supplementation reduced oocyte reactive oxygen species (ROS) and boosted the cleavage rate of embryos during HS conditions [7].

Exposure to high temperatures for a short time can negatively impact oocyte quality [8–10]. During HS, mitochondrial activity rises to exceed capacity, leading to increased ROS production. Mitochondria are the main source of ROS formation as a by-product of the electron transfer process complexes I, II, and III [11]. Oxidative stress can occur if ROS production exceeds the antioxidant capacity. Under conditions of oxidative stress, mitochondrial permeability increases and causes excess ROS production, cessation of adenosine triphosphate (ATP) synthesis, and a decrease in glutathione (GSH) [12]. The oocyte response to the effects of HS can increase the amount of ROS, which can reduce antioxidant levels both *in vitro* and *in vivo* [13].

This study used Zebrafish (*Danio rerio*) as a research animal model because they have high genetic similarities with mammals. This high genetic similarity can be used for biomedical research and developmental studies in vertebrates, such as studies of development, disease, and behavior in humans [14]. Zebrafish are small freshwater teleosts that are transparent, fast-growing and exhibit highly conserved developmental programs. Moreover, the housing and husbandry space required for this species is small and inexpensive [15].

Indonesia is known as a producer of local and national types of oranges throughout the archipelago, from Sabang to Merauke, which can be developed to meet the vitamin and mineral needs of the community [16]. Oranges also contain active ingredients such as polyphenolic compounds (phenolic acids and flavonoids), which have anti-inflammatory and antioxidant properties [17]. Orange peel (OP) composition includes lipids, carbohydrates (glucose, fructose, and sucrose), organic acids (citric, malic, malonic, and oxalic), carbohydrate polymers (cellulose, hemicellulose, and pectin), enzymes (pectin esterase, phosphatase, and peroxidase), flavonoids, oils, essentials, and pigments. OP contains phenolic compounds that act as antioxidants. Flavonoids are important natural phenolics because they have radical scavenging properties. OP contains polyphenol compounds such as naringin, routine, hesperidin, and quercetin in large quantities [18].

Flavonoid antioxidants function as hydroxyl radical scavengers. Antioxidants work by delaying, preventing, and eliminating oxidative damage to molecules. In general, flavonoids play a role in warding off free radicals, binding metals, and suppressing enzymes that induce the formation of free radicals and stimulating internal antioxidant enzymes. Flavonoids can bind free radicals by donating hydrogen atoms or transferring electrons [19].

The optimum temperature for Zebrafish ranges from 24°C to 28°C [20], while at 32°C, Zebrafish exhibit thermal sensitivity [21], and 34°C represents thermal stress. In this study, we hypothesized that HS induction (32°C and 34°C) impacted diameter, survival rate, and germinal vesicle breakdown (GVBD) of zebrafish oocytes (ZOs) in which these negative effects would be overcome with OP supplementation (328.58 ppm), to provide an alternative solution to face the detrimental effects of global warming focusing on female fertility.

## Materials and Methods

### Ethical approval

All protocols were approved by the Institutional Animal Care and Use Committee of Universitas Brawijaya, Malang, Indonesia (No.201-KEP-UB-2023).

### Study period and location

The study was conducted during July and August 2023 at the Veterinary Embryology Laboratory and Veterinary Parasitology Laboratory, Faculty of Veterinary Medicine, Universitas Brawijaya. The OP extract was processed at the Matera Medica Laboratory, Batu City.

### Chemicals and reagents

The materials used in this research were 20 Zebrafish broodstock (Fish Reproduction Laboratory, Faculty of Fisheries and Marine Sciences, Universitas Brawijaya), stage 3 ZO (n = 1080), OP extract (Dau, Malang, Indonesia; north latitude: −7.939081365604272, south latitude: 112.55396061058408; temperature: 18.4°C–32.7°C; humid: 79–86%), 96% ethanol (OneMed, Indonesia), distilled water (OneMed, Indonesia), *in vitr*o maturation (IVM) media, 0.1% trypan blue (TB) (HiMedia, India), and phosphate buffer sulfate (HiMedia).

### Oocyte recovery

ZO was collected after necropsy using a lower abdominal incision. The selected final stage (Phase III) of the ZO (n = 1080) was then treated with 4 μL of OP extract (not treated/control) under HS: 32°C (Heat stress 32°C solution/Heat stress 32°C orange peel [HS32S/HS32O]) and 34°C (Heat stress 34°C solution/Heat stress 34°C orange peel [HS34S/HS34O]); and NHS: 28°C (Non-heat stress solution/Non-heat stress orange peel [NHSS/NHSO]) during maturation.

The HS group was incubated in a Water bath (Memmert^®^, Germany) at 32°C and 34°C for 1 h. Meanwhile, the NHS group was incubated in an incubator at 28°C.

IVM media (2 mL) was pipetted into six-well plates. Each well contains two oocytes in five wells and 10 oocytes in 1 well. Treatment with extracts and HS were divided into six groups: HS32O, HS32S, HS34S, NHSO, and NHSS. In each group given the OP treatment, 4 μL was given in each well of the six-well plate.

### Preparation of the OP extract

The peels were initially cleaned with running water to remove any dirt. Subsequently, they were dried under sunlight. Next, the dried peels were pulverized using a blender until becoming powder. The resulting powder was weighed and dissolved in 96% ethanol solvent (ratio: 1:5) at room temperature (28°C) for 3 × 24 h. Afterward, it was filtered and evaporated using a rotary evaporator (40°C; 3 h) to remove any remaining solvent [22]. Moreover, the flavonoid concentration on OP extract was determined using an ultra-violet visible spectrophotometer (Shimadzu UV-Vis 1800, Japan) (absorbance: 2.4870), yielding 328.58 ppm of flavonoid concentration.

OP extract with 90% ethanol produced the strongest antioxidant activity compared with other extracts (IC_50_ 348.2 ppm). Increasing the time from 10 min to 30 min in extraction with 80% and 85% ethanol resulted in a decrease in the IC_50_ value (increased antioxidant activity) but not in extraction with 90% ethanol [23].

### Making IVM media

IVM is a phase maturation medium from the immature or germinal vesicle (GV) phase to the metaphase II stage, which can be used to observe the mechanisms of oogenesis and folliculogenesis. The maturation process in fish is characterized by the resumption of meiosis and vesicle breakdown (GVBD) [24, 25].

Selected third-stage oocytes were placed in IVM media containing 3.74 g sodium chloride, 0.32 g potassium chloride, 0.16 g calcium chloride, 0.10 g sodium phosphate monobasic dihydrate, 0.16 g magnesium sulfate heptahydrate, 0.40 g glucose, and 0.008 g phenol red, penicillin, and streptomycin sulfate. All these ingredients were dissolved in 1 L of distilled water and added sodium bicarbonate (1 mol/L) until it reached a pH of 7.5. Penicillin (200,000 IU) and streptomycin sulfate (200 mg) were added to prevent medium contamination. The oocytes were placed in an incubator set at a temperature of 30°C for 4 h [26].

### Observation of the research parameters

Observations were carried out by transferring oocytes from a 6-well plate to the object glass concave one by one using a plastic pipette and then observed using a binocular microscope (Olympus CKX53, Japan) and OptiLab (Adavance Plus, Indonesia), which was calibrated using a micrometer. Next, the diameter data are processed using ImageJ software to measure the average area of oocytes. After obtaining data in the form of numbers from ImageJ, the data are processed using Microsoft Excel to calculate the diameter of each oocyte. Observation of survival and GVBD rate of oocytes was performed by moving the oocytes to a six-well plate. Following this, 0.4 mL was stained TB 0.1% for 40 s and then washed to remove the staining residue. The coloring was performed with TB 0.1% for 40 s in 1 mL for each well and rinsed using distilled water 3 times [27, 28].

Oocytes formed during the prophase of meiosis I contain a large nucleus surrounded by a nuclear membrane known as GV. During oocyte maturation, there is a chromatin maturation process and GV breakdown, known as GVBD. GVBD, or the disappearance of the oocyte nuclear membrane, is a sign that an oocyte has successfully matured [29]. TB is a synthetic dye commonly used to assess membrane integrity of cells [30]. The mechanism of TB staining is based on the negatively charged dye molecule, which cannot interact with live cells. When the oocytes do not mature yet, the GV will not break down and the TB cannot be absorbed by the GV, which makes it colorless of the GV. By contrast, when the GV was break down (GVBD), TB will bind with intracellular proteins and resulting in blue color [31, 32]. The survival and GVBD rates were observed under a microscope with 40× magnification.

### Statistical analysis

ZO observations were performed using quantitative analysis measuring the diameter of the oocyte, survival rate, and GVBD rate. Data were processed using IBM SPSS Statistics 23 software (IBM Corp., NY, USA) and two-way analysis of variance. Kruskal–Wallis test was used to determine the significant average difference in the influence of the independent variable on the dependent variable using the Statistical Product and Service Solutions program. Significance was recorded at p < 0.05.

## Results

### Results of OP extract treatment on the ZO diameter

**Figure-1:**
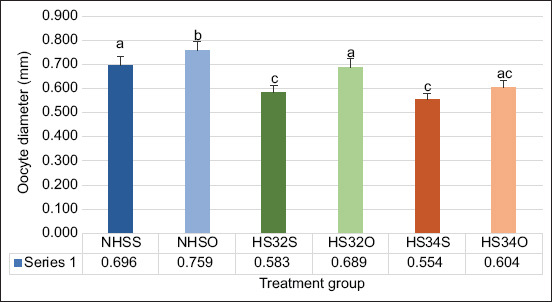
Comparison of orange peel extract on oocyte diameter at different temperatures (NHSS=Non-heat stress Solution, NHSO=Non-heat stress orange peel, HS32S=Heat stress 32°C solution, HS32O=Heat stress 32°C orange peel, HS34S=Heat stress 34°C solution, HS34O=Heat stress 34°C orange peel). Data were analyzed using a two-way analysis of variance and Kruskal–Wallis (p < 0.05).

**Figure-2:**
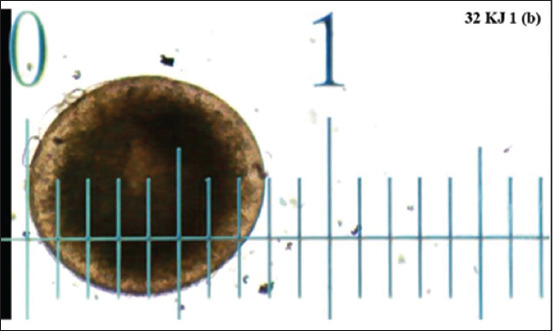
Illustration of diameter measurements of the zebrafish oocyte with the ImageJ application (Personal Documentation, 2023).

The diameter of the NHSO group (0.759 ± 0.01) showed a significant (p < 0.05) increase in diameter compared with NHSS (0.696 ± 0.02). In the HS32S group, the temperature (0.583 ± 0.02) had a significant difference from the HS32O group (0.689 ± 0.02). In the HS34S group, the temperature (0.554 ± 0.02) had a significant difference from the HS34O group (0.604 ± 0.02). The HS group obtained the lowest values in the presence or absence of OP treatment (Figure-1). The illustration of the diameter measurement is presented in Figure-2.

### Results of OP extract treatment on the survival rate of ZOs

**Figure-3:**
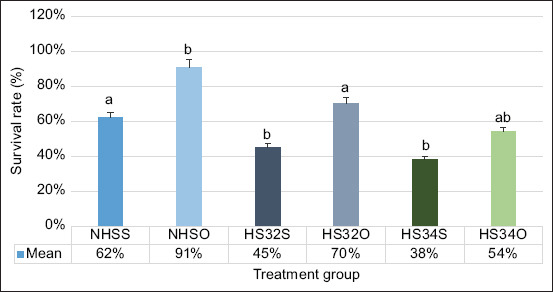
The results of the graphic survival rate of the zebrafish oocyte at different temperatures (NHSS=Non-heat stress solution, NHSO=Non-heat stress orange peel, HS32S=Heat stress 32°C solution, HS32O=Heat stress 32°C orange peel, HS34S=Heat stress 34°C solution, HS34O=Heat stress 34°C orange peel). Data were analyzed using a two-way analysis of variance and Kruskal–Wallis (p < 0.05).

**Figure-4:**
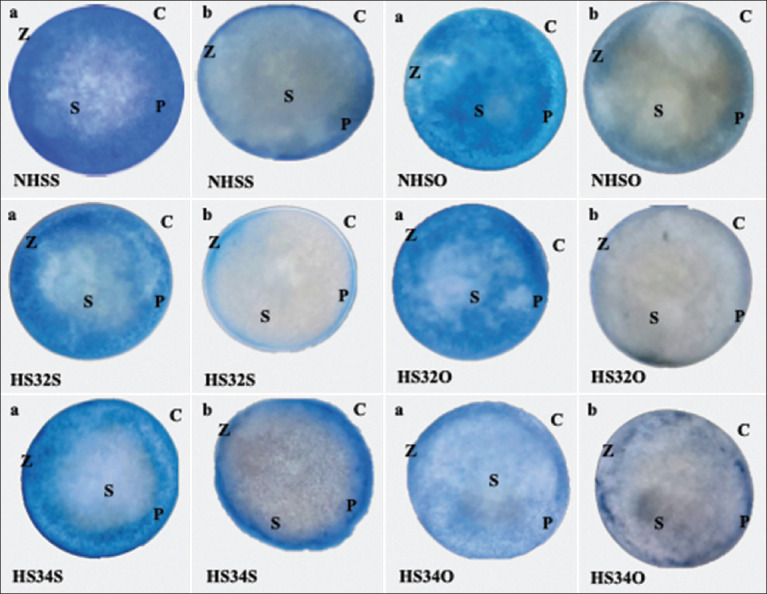
Zebrafish oocyte was exposed to 0.1% trypan blue (TB) staining. (a) Live oocytes are not marked blue and (b) dead oocytes are marked blue by TB. Z=Zona pellucida, C=Corona radiate, P=Perivitelline space, S=Cytoplasm (Personal Documentation, 2023).

The percentage survival rate obtained from treatment group (NHSO [91% ± 5%], HS32O [70% ± 3%], and HS34O [54% ± 3%]) was significantly (p < 0.05) higher than those obtain from non-treatment group (NHSS [62% ± 6%], HS32S [45% ± 6%], and HS34S [38% ± 5%]) (Figure-3). The microscopic image of 0.1% TB staining in ZOs used to calculate the survival rate is shown in Figure-4.

### Results of OP extract treatment on the GVBD of ZOs

**Figure-5:**
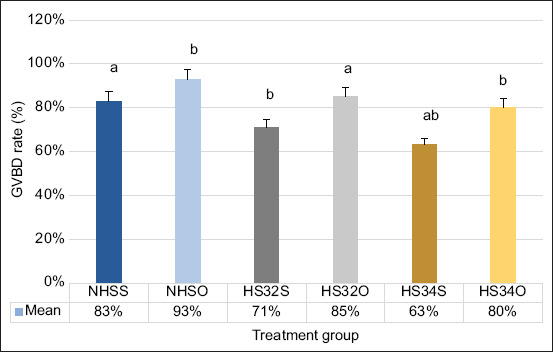
Graphic diagram of test results for calculating the mean germinal vesicle breakdown rate of zebrafish oocytes in each treatment group. (NHSS=Non-heat stress solution, NHSO=Non-heat stress orange peel; HS32S=Heat stress 32°C solution; HS32O=Heat stress 32°C orange peel; HS34S=Heat stress 34°C solution; HS34O=Heat stress 34°C orange peel; different notations indicate significant differences between treatments at each temperature).

**Figure-6:**
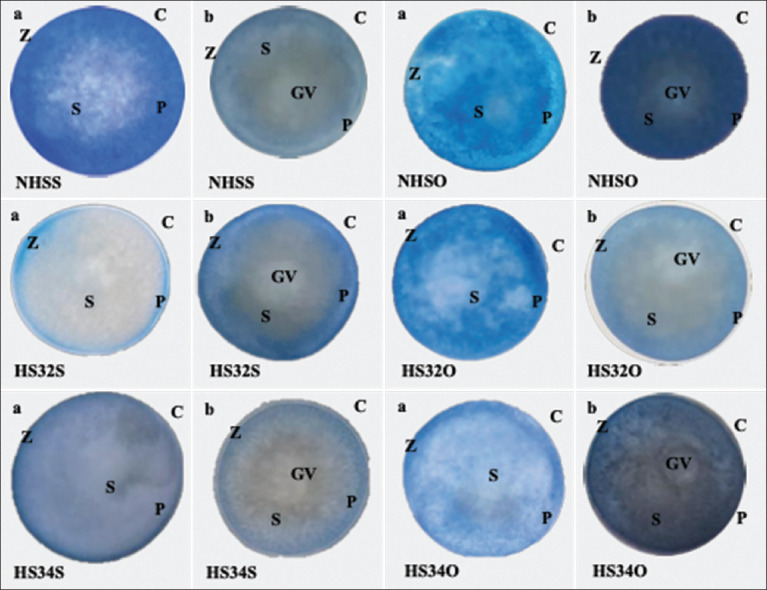
Microscopic image of trypan blue staining on Zebrafish oocytes to calculate the germinal vesicle breakdown (GVBD) rate with 40× magnification. (a) Oocytes experiencing GVBD (mature) are characterized by the fusion of the nucleus and (b) Germinal vesicle oocytes are characterized by the nucleus remaining in the oocyte. The arrow shows the nucleus of the oocyte. C=Corona radiata, Z=Zona pellucida, P=Perivitelline space, S=Cytoplasm, GV=Germinal vesicle (Personal Documentation, 2023).

The percentage of GVBD rate obtained from treatment group (NHSO [93% ± 3%], HS32O [85% ± 2%], and HS34O [80% ± 2%]) was significantly (p < 0.05) higher than those obtain from non-treatment group (NHSS [83% ± 3%], HS32S [71% ± 6%], and HS34S [63% ± 3%]) (Figure-5). The microscopic image of TB staining on ZOs used to calculate the GVBD rate is presented in Figure-6.

## Discussion

This study showed that treatment with maturation media significantly increased diameter, survival rate, and GVBD rate compared with the non-treatment group. In one study, oocytes in HS conditions experienced disruption during maturation, resulting in a small oocyte size [23]. Heat exposure in GV-stage oocytes can reduce oocyte developmental competence [25, 33]. Stress conditions in rainbow trout during the early vitellogenesis phase resulted in smaller fish eggs than normal. In addition, according to Kawano *et al*. [8], the average oocyte diameter in cows after IVM was significantly smaller in the HS group than in the control group. The presence of impaired oocyte growth indicates a decrease in the ability of oocytes to develop due to heat exposure. This research is in accordance with the literature, which states that Zebrafish embryos seem to develop normally in the temperature range of 25°C–33°C. Long exposure to high or low temperatures may cause abnormalities [34]. This shows that the optimal temperature and OP treatment in the NHS group can increase the diameter of ZO.

Oxidative stress can occur if ROS production exceeds the antioxidant capacity. Under conditions of oxidative stress, mitochondrial permeability increases and causes excess ROS production, cessation of ATP synthesis, and a decrease in GSH [12]. This research agrees with Alix *et al*. [35], who stated that higher temperatures than the optimal temperature have a negative impact on developmental success, such as reduced survival, hatching rate, and increased opportunities for abnormal development (for example, abnormal cell division), as well as the rate of deformity of newly hatched larvae, regardless of the duration or time of thermal exposure. Increasing temperature during the vitellogenesis phase has been shown to decrease fertility and egg survival rates in salmon [36]. This shows that the NHSO group (91% ± 5%) proven at optimal temperature with treatment can increase the oocyte survival rate. This is because the oocyte membrane does not experience changes in permeability due to ROS, so it can prevent TB dye from entering the cell. The enhanced survival rate obtained from the results of live oocytes was characterized by the oocyte not being stained by TB. In addition, treatment with the OP extract showed an increase in the HS and NHS groups. The NHS group had optimal development compared with the HS group because the NHS group had no heat exposure at all.

Increased free radicals in bovine oocytes can stop meiosis, lead to poor oocyte quality, and lower embryo development [13]. Excess ROS can affect the ability of oocytes, especially in the maturation of the nucleus and cytoplasm [34]. The research carried out was in accordance with Rakha *et al*. [13], who found that HS can change the nuclear and cytoskeletal components of oocytes, which can inhibit oocyte development. Oocytes in the maturation phase will continue meiosis to metaphase II, which is accompanied by a maturation process in the nucleus and cytoplasm of the oocyte. This process is called oocyte maturation, which occurs before ovulation and is a condition for successful fertilization, consisting of the breakdown of the GV (GVBD), chromosome condensation, meiotic spindle assembly, and the formation of the first polar body [19]. The NHS group’s optimal temperature and OP treatment, without heat exposure, enhance the GVBD rate of ZO. The OP extract led to an increase in HS and NHS groups. The NHS group outperformed the HS group as they did not experience any heat exposure. Antioxidants derived from OP effectively reduce oxidative stress by inhibiting ROS molecules, enhancing the quality of cultured cells [37].

## Conclusion

The study reveals that oocytes undergoing HS exhibit increased oxidative stress. The OP extract treatment group under NHS conditions experienced an increase in diameter, survival rate, and GVBD rate. Under HS and NHS conditions, flavonoid antioxidants from OP extract waste enhance ZO maturation and development. However, it is necessary to conduct molecular studies regarding the mechanism of flavonoids in OP extract to counteract the presence of ROS caused by HS induction.

## Authors’ Contributions

HSAT and GR: Planned and designed the study. MAL and AKA: Organized and supervised the fieldwork. HSAT, GR, BKL, and AGS: Drafted and reviewed the manuscript. HSAT, GR, BKL, and AGS: Analyzed data and developed the main body text. GR and AGS: Addressed most of the discussion. HSAT, GR, BKL, and AGS: Revised the manuscript. All authors have read, reviewed, and approved the final manuscript.
